# Reproductive Toxicity of Furfural Acetone in *Meloidogyne incognita* and *Caenorhabditis elegans*

**DOI:** 10.3390/cells11030401

**Published:** 2022-01-25

**Authors:** Wanli Cheng, Xue Yang, Hua Xue, Dian Huang, Minmin Cai, Feng Huang, Longyu Zheng, Ziniu Yu, Jibin Zhang

**Affiliations:** State Key Laboratory of Agricultural Microbiology and National Engineering Research Center of Microbial Pesticides, College of Life Science and Technology, Huazhong Agricultural University, Wuhan 430070, China; chengwanli@mail.hzau.edu.cn (W.C.); sj1501yx@webmail.hzau.edu.cn (X.Y.); 2020304110160@webmail.hzau.edu.cn (H.X.); huangdian@mail.hzau.edu.cn (D.H.); cmm114@mail.hzau.edu.cn (M.C.); fenghuang@mail.hzau.edu.cn (F.H.); ly.zheng@mail.hzau.edu.cn (L.Z.); yz41@mail.hzau.edu.cn (Z.Y.)

**Keywords:** furfural acetone, *Meloidogyne incognita*, *Caenorhabditis elegans*, reproductive toxicity, germ-cell apoptosis

## Abstract

Furfural acetone (FAc) is a promising alternative to currently available nematicides, and it exhibits equivalent control efficiency on root-knot nematodes with avermectin in fields. However, its effect on the reproduction of root-knot nematode is poorly understood. In this study, the natural metabolite FAc was found to exhibit reproductive toxicity on *Meloidogyne incognita* and *Caenorhabditis elegans*. The number of germ cells of *C. elegans* was observed to decrease after exposure to FAc, with a reduction of 59.9% at a dose of 200 mg/L. FAc in various concentrations induced the germ-cell apoptosis of *C. elegans*, with an increase over six-fold in the number of apoptotic germ cells at 200 mg/L. These findings suggested that FAc decreased the brood size of nematode by inducing germ-cell apoptosis. Moreover, FAc-induced germ-cell apoptosis was suppressed by the mutation of gene *hus-1*, *clk-2*, *cep-1*, *egl-1*, *ced-3*, *ced-4*, or *ced-9*. The expression of genes *spo-11*, *cep-1*, and *egl-1* in *C. elegans* was increased significantly after FAc treatment. Taken together, these results indicate that nematode exposure to FAc might inflict DNA damage through protein SPO-11, activate CEP-1 and EGL-1, and induce the core apoptosis pathway to cause germ-cell apoptosis, resulting in decreased brood size of *C. elegans*.

## 1. Introduction

Plant-parasitic nematodes (PPNs) are a category of the most serious plant parasites and include over 4100 different species [1]. PPNs infect the plant host by using their stylets or mouth spears, and affect the nutrient absorption and growth of crops, resulting in serious reduction in crop production and threatening the global food security [2]. To date, diseases caused by PPNs result in more than USD 150 billion in economic losses worldwide each year [3], and more than half of the economic losses are due to root-knot nematodes (RKNs) [4]. RKNs are considered to be one of the most damaging nematode groups since they can infect most of cultivated plant species in the world [5]. RKNs have strong reproductive ability; a mature female can lay up to 1000 eggs in an egg mass [2]. The characteristic of large brood size confers difficulty in controlling RKNs.

Chemical nematicides currently remain the primary means to manage RKN in agricultural crops [6]. However, the long-term and excessive use of chemical nematicides increases nematode resistance and risks to the ecological environment [6,7]. Thus, novel, effective, and environment-friendly nematicides are urgently needed. Biological control is a promising environmentally suitable approach to reduce nematode losses. Many microorganisms [8,9,10], plants [11,12,13], or their metabolites [14,15] reportedly possess nematicidal activity. However, most have poor practical application efficiency in field. Furfural acetone (FAc), also known as 4-(2-furyl)-3-buten-2-one, is a volatile organic compound produced by microbes and plants [16,17]. FAc reportedly exerts multiple effects on *M. incognita* [18,19] and achieves equivalent efficiency against RKNs with the commercial nematicide avermectin in the field [19]. FAc was recognized as a type of flavoring agent and food additive in 2019 [20]. All of these characteristics of FAc provide a compelling basis for considering it as a promising alternative to currently available nematicides.

*Caenorhabditis elegans* is a multicellular worm extensively used as a biomedical model organism for the ecotoxicological studies of toxicants and reproductive studies [21,22,23,24]. The powerful advantage of studying the germ line of *C. elegans* over other animals is that all stages of meiosis can be observed at once [25]. Many active compounds have reproductive toxicity [22,23], but the molecular mechanism remains poorly understood. Germ-cell apoptosis in *C. elegans* can induce oocyte cell death, leading to nematode reproductive toxicity [26,27]. In *C. elegans*, the p53-like protein CEP-1 regulates the apoptotic response to DNA damage during oogenesis [27,28]. When DNA double-strand breaks are induced by SPO-11, ionizing radiation or other stress and persist into late pachytene, the DNA damage checkpoint proteins (such as RAD-5, CLK-2, or HUS-1) induce the activation of protein CEP-1 [27]. CEP-1 transcriptionally induces protein EGL-1 or CED-13 that alleviate the core apoptotic response pathway (including CED-9, CED-4, and CED-3) and thus promoting germ cell apoptosis [26,27,28].

In the present study, FAc was found to have reproductive toxicity to *M. incognita* in a pot experiment. We then used the model nematode *C. elegans* to investigate the molecular mechanism of FAc in inhibiting nematode reproduction.

## 2. Materials and Methods

### 2.1. Chemical

Furfural acetone (FAc, purity ≥ 98%) used in this study was purchased from TCI (Tokyo, Japan). FAc was dissolved in sterile water or S medium to determine its effects on nematodes.

### 2.2. Nematodes

*Meloidogyne incognita* individuals were cultivated on tomato (ZhongShu No. 4) roots grown in a greenhouse at 25 °C. The egg masses of *M. incognita* were peeled from the infected root galls with needles and washed three times in sterile water. The egg masses were then placed in a 2 mL centrifuge tube and immersed in sterile water at 20 °C for 72 h. J2 juveniles hatched within 3 days were collected and used immediately.

All *C. elegans* strains were cultured and maintained at 20 °C on nematode growth medium and fed with *Escherichia coli* OP50. N2 Bristol strain (wild-type), *qIs56 [lag-2::gfp]* (JK2868), *hus-1(op241)*, *clk-2(qm37)*, *cep-1(ep347)*, *egl-1(n987)*, *ced-3(n717)*, *ced-4(n1894)*, and *ced-9(n1950)* were provided by the *Caenorhabditis* Genetics Center. Adult *C. elegans* were treated with 5% sodium hypochlorite: 5 M sodium hydroxide (2:1, *v*/*v*), and embryo hatching on NGM plates overnight to obtain synchronized L_1_ worms. Synchronized L_4_ or young-adult hermaphrodites was obtained by feeding synchronized L_1_ worms with *E. coli* OP50 and used in this study.

### 2.3. Pot Experiment

Tomato (ZhongShu No. 4) seeds were soaked in 2% sodium hypochlorite for 5 min for surface disinfection, rinsed five times in sterile water, and then incubated in organic matter for 20 days. Tomato plants of the same size were selected to use in pot experiments. Each plastic round pot (7 cm × 7 cm × 9 cm) was filled with 100 g of sterile soil mixture (sand: field soil: organic matter, 1:1:1). Each pot was transplanted with a tomato seedling and incubated in the greenhouse at 24–26 °C; about 1000 freshly hatched J2 juveniles of *M. incognita* were inoculated into the rhizosphere soil of each seedling after 2 days. FAc and avermectin (Tenov, Weifang, China) were dissolved in water, and the tomato seedlings were irrigated around the roots with either 5 mL of FAc solution (1 or 2 mg/pot), avermectin solution (1 mg/pot), or water alone (control group) 2 days after the nematode inoculation. Each treatment had five replicates. After 60 days, tomato plants in each group were separated carefully from the soil, and the fresh weights of roots and the number of galls on roots were recorded after being washed in a gentle flow of water.

To estimate the number of nematodes in tomato roots, roots were cut into 2 cm-long pieces, lysed in 1% sodium hypochlorite for 15 min, and crushed in a bowl for 3 min. The roots and solution were passed through two different filters with pore sizes of 45 and 25 μm, and the nematodes were collected from the 25 μm filter by spraying with sterile water [5]. The egg masses of *M. incognita* were peeled from the infected root galls with needles, and washed three times with sterile water. A total of three egg masses for each plant were randomly selected to count the average number of eggs in each egg mass. The egg mass was lysed with 1% sodium hypochlorite solution for 5 min, and the solution was passed in turn through 3 different filters with pore sizes of 74, 45, and 25 μm. The eggs of *M. incognita* were collected from the 25 μm filter by spraying with sterile water [29]. The soil attached to the tomato root was collected; a total of 5 g of the soil in each pot was selected to detect the population density of nematodes in the soil. These nematodes in the soil were collected into 90 mm Petri dishes by sugar centrifugation flotation [30] and counted under an inverted microscope (Olympus IX73, Olympus, Tokyo, Japan).

### 2.4. Effect of FAc on Nematode Reproduction

The effect of FAc on nematode reproduction was determined using 96-well plates as previously described [19,31]. To each well was added a single synchronized L_4_ hermaphrodites, 5 μL of *E. coli* OP50 cultured in S medium (OD600 = 2.0), and 115 μL of FAc solution dissolved in S medium [31]. The S medium served as a control, and each concentration of FAc was tested with five wells. The plates were maintained at 20 °C for 72 h, and the total number of eggs hatched by a worm was counted under an inverted microscope (Olympus IX73, Olympus, Tokyo, Japan). The experiment was repeated three times.

### 2.5. Nematode Germ Cells Determination

The number of germ cells in the gonad was counted after staining with DAPI as previously described [32,33]. About 40 synchronized L_4_ wild-type *C. elegans* were transferred to 200 μL of FAc solution (dissolved in sterile water) for 24 h, and at least 10 worms in each group were picked out and suspended in 1 μL of sterile water. After fixing with Carnoy’s fixative (60% ethanol, 30% chloroform, and 10% glacial acid), air drying, and staining with a drop of 2 μg/mL DAPI (MERCK, Darmstadt, Germany) solution (dissolved in M9 buffer), the number of germ cells in the gonad were counted under a confocal laser scanning microscope (Nikon A1HD25, Nikon, Tokyo, Japan).

### 2.6. Nematode Gonadal Development Assay

Larval gonadal development assays were conducted as previously described [34]. Approximately 40 L_4_ larvae of JK2868 nematodes were exposed to FAc in various concentrations for 24 h and then washed three times in sterile water. Worms were transferred to slides and captured with a fluorescence microscope (Nikon Ti-U, Nikon, Tokyo, Japan). Distal tip cells (DTCs) of transgenic nematode *qIs56 [lag-2::gfp]* (JK2868) were labeled with green fluorescence, and the fluorescence intensity of green fluorescent protein was evaluated by quantitative analysis using Image J1.33 software (NIH, Bethesda, MD, USA). At least 11 nematodes were tested in each treatment group.

### 2.7. Apoptosis Assays

Apoptosis assays were conducted as previously described [22,23] with some modifications. About 30 synchronized young-adult *C. elegans* were transferred into each well of 96-well plates containing sterile water with or without FAc for 24 h at 20 °C. The exposed worms were washed with sterile water three times and preincubated for 1 h at 20 °C in M9 buffer containing *E. coli* OP50 and 30 mg/mL acridine orange (AO, MERCK, Darmstadt, Germany). Nematodes were then washed with sterile water three times and recovered for 30 min on nematode growth-medium plates. Nematodes in each treatment were transferred onto a slide with 60 mg/mL sodium azide in M9 buffer, and the number of apoptotic cells in a gonad arm was counted by using a fluorescent microscope (Olympus BX63, Olympus, Tokyo, Japan). The test was repeated at least five times.

### 2.8. Quantitative Real-Time PCR

The total RNA of wild-type *C. elegans* was isolated by using an RNAsimple Total RNA Kit (TIANGEN, Beijing, China) according to the manufacturer’s instructions and used to synthesize 20 μL of cDNA according to kit protocols by using a BeyoRTTM II First Strand cDNA Synthesis Kit (Beyotime, Shanghai, China). Quantitative real-time PCR (qPCR) was performed in 10 μL reactions with 0.2 μM final primer concentrations according to the kit protocols of AceQ^®^ qPCR SYBR^®^ Green Master Mix (2×, Low ROX) (Vazyme, Nanjing, China). Cycling conditions (initial denaturation at 95 °C for 5 min; followed by 40 cycles of 15 s at 95 °C, 30 s at 60 °C, and 15 s at 95 °C; 60 s at 60 °C, and 15 s at 95 °C) were used to analyze the melting curve and measure the specificity in each reaction tube. For all qPCR experiments, each biological replicate was measured in three technical replicates and normalized to the control gene *pmp-3* [35], which was expressed stably in *C. elegans*. The ∆∆Ct method was used to calculate mRNA levels [36]. The experiment was repeated three times at least. The primers used for qPCR were as follows: *pmp-3*: 5′- CCA GAT CAA CGT CTA ACC CAA -3′ (F), 5′- GCG GGA CCA ATC CAA CC -3′ (R); *egl-1*: 5′- GTC TCA GGA CTT CTC CTC GTG -3′ (F), 5′- GAG CAT CGA AGT CAT CGC AC -3′ (R); *cep-1*: 5′- TGG GAT GTC TAG TGC CGA TTC -3′ (F), 5′- TCT CGT TCA GTA TGA CTT CGA CA -3′ (R); *spo-11*: 5′- ATG TGC GGA CAG GAG TA -3′ (F), 5′- TTG TGA ATC TTC GTG GT -3′ (R).

### 2.9. Statistical Analysis

All data were analyzed by using SPSS version 22.0 software (SPSS, Chicago, IL, USA) and shown as the mean ± standard error. The significance of the differences between two groups was assessed by two-tailed unpaired Student’s t test, and *p* < 0.05 was considered significant. Least significant difference (LSD) test was used to test for significant differences among different treatments at *p* = 0.05 and different lowercase letters indicated significant difference among treatments.

## 3. Results

### 3.1. Efficiency of FAc against M. incognita in Pots

**Figure 1:**
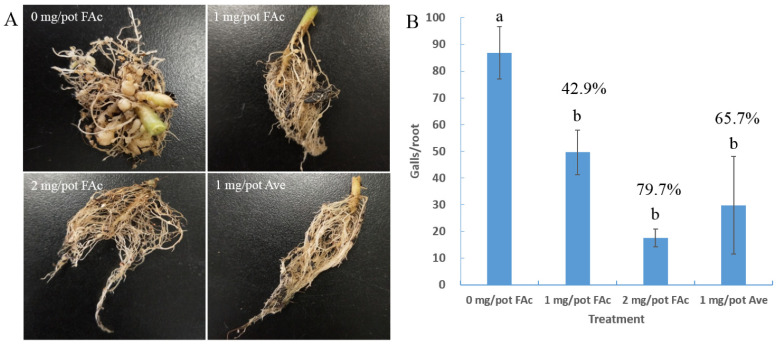
Control efficiency of FAc against *M. incognita* in pots. (**A**) Roots of tomato plants after treatment with FAc or avermectin (Ave). (**B**) Control efficiency of FAc and Ave against *M. incognita* in pots. Data are shown as the mean ± standard error (*n* = 5), and different lowercase letters indicate significant difference among treatments by LSD test (*p* < 0.05). The numbers above the lowercase letters indicate the control effect of each group compared with the control. Control effect (%) = ((galls in control − galls in treated)/galls in control) × 100.

FAc solution was irrigated into the rhizosphere of tomato plants to evaluate its efficiency against *M. incognita* in pots. RKNs infected and formed numerous large root galls in the tomatoes of the control group (0 mg/pot FAc), whereas fewer galls were observed on tomato roots in the treatment groups (Figure 1A). The number of tomato root galls significantly decreased in the FAc treatment groups compared with that of control, with control effects of 42.9% and 79.7% at doses of 1 and 2 mg/pot, respectively. No significant difference was observed from that (65. 7%) of avermectin at a dose of 1 mg/pot (Figure 1B). These results were consistent with previous studies [18,19], indicating the stable RKN control efficiency of FAc.

To better evaluate the efficiency of FAc on RKNs in pots, the number of nematodes in the tomato roots and rhizosphere soil was also recorded. Compared with the control, the number of nematodes in tomato roots after treatment with FAc decreased significantly. The number of nematodes in 1 g of roots decreased significantly from 4373 in the control group to 2326 and 1775 in the FAc treated groups at doses of 1 and 2 mg/pot, respectively (Table 1). Similarly, compared with control group, the number of nematodes in the soil decreased significantly after FAc application, with nematode reductions of 49.6% and 57.1% at doses of 1 and 2 mg/pot, respectively (Table 1). In summary, FAc exhibited equivalent anti-nematode activity to avermectin in pots, as it can significantly reduce the number of galls on tomato roots and the number of *M. incognita* in tomato roots and soil. Thus, FAc is a promising alternative to currently available nematicides.

### 3.2. FAc Reduced the Brood Size of Nematode

**Figure 2:**
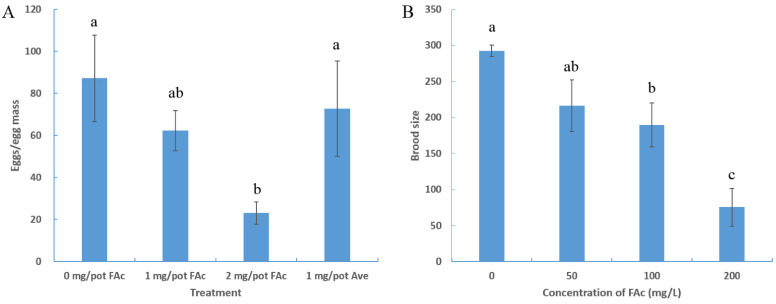
FAc inhibited nematode reproduction. (**A**) Number of *M. incognita* eggs in an egg mass on tomato roots after treatment with FAc or avermectin (Ave); data are shown as the mean ± standard error (*n* = 5). (**B**) Brood size of *C. elegans* immersed in FAc solution in various concentrations for 72 h. Data are shown as the mean ± standard error (*n* = 3), and different lowercase letters indicate significant difference among treatments by LSD test (*p* < 0.05).

After separating plant roots from the pot, the number of eggs in an egg mass of each group was counted. No significant difference in the number of eggs in an egg mass existed between the control group and the 1 mg/pot avermectin treatment group. The number of eggs/egg mass significantly decreased after treatment with FAc compared with the control group, with an egg reduction of 73.6% at a dose of 2 mg/pot (Figure 2A). This finding suggested that FAc may inhibit the reproduction of *M. incognita*. Accordingly, the model nematode *C. elegans* was used to verify whether FAc could inhibit nematode reproduction further. As shown in Figure 2B, FAc also significantly affected the reproduction of *C. elegans*. Increased concentration of FAc caused a coordinated decline in the brood size of *C. elegans*, with a 74.6% decrease in brood size after exposure to 200 mg/L FAc. These results suggested that nematode exposure to FAc lead to decrease in brood size, indicating the reproductive toxicity of FAc on nematodes.

### 3.3. FAc Exposure Induced Germ-Cell Reduction of C. elegans

**Figure 3:**
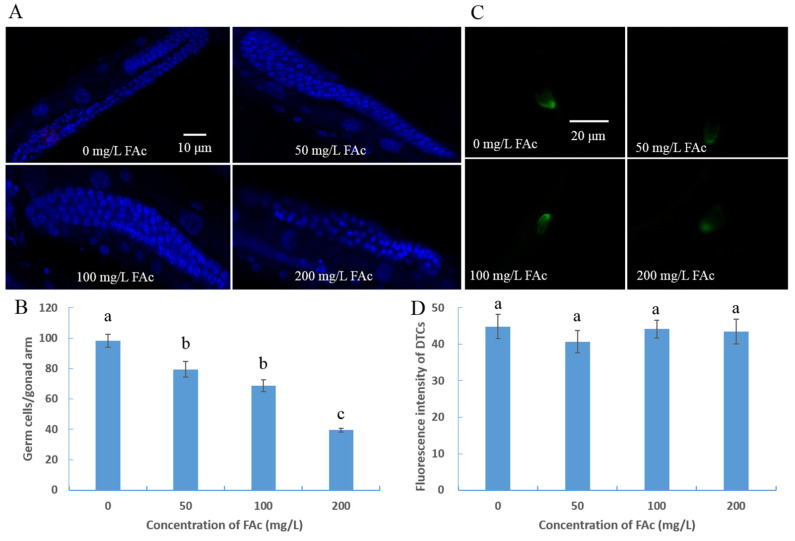
FAc-induced nematode germ-cell reduction. (**A**) Images of germ cells of *C. elegans*, wild-type L4 worms were treated with FAc in different concentrations for 24 h, and stained by using DAPI. The bar denotes 10 μm. (**B**) Number of germ cells of *C. elegans* after exposure to FAc for 24 h; data are shown as mean ± standard error (*n* ≥ 10). (**C**) Images of distal tip cells (DTCs) of JK2868 nematodes after exposure to different concentrations of FAc. The bar denotes 20 μm. (**D**) Fluorescence intensity of DTCs of JK2868 nematodes exposed to different concentrations of FAc. Data are shown as the mean ± standard error (*n* ≥ 11), and different lowercase letters indicate significant difference among treatments by LSD test (*p* < 0.05).

After confirming that FAc could reduce the brood size of nematodes, we explored whether the reduction in brood size was caused by a lower total number of germ cells. Because adult *M. incognita* females live in plant roots and are difficult to culture in vitro, the model nematode *C. elegans* was used to investigate further the reproductive-toxicity molecular mechanism of FAc on nematodes. The germ cells of *C. elegans* exposed to FAc were counted after staining with DAPI. In *C. elegans*, after exposure to FAc for 24 h, the number of germ cells in a nematode decreased with increased concentration of FAc (Figure 3A). When nematodes were immersed in 200 mg/L FAc solutions, the number of germ cells per gonad arm reduced from 98.4 in the control group (0 mg/L) to 39.5, with a reduction of 59.9% (Figure 3B). These results demonstrated that the reduction in brood size of nematodes under exposure to FAc was due to the decrease in the number of germ cells.

Whether the reduction of germ cells of *C. elegans* is caused by affecting gonadal development was studied further. The DTCs act as the germ line stem cell niche, and each DTC caps a pool of germ stem cells [37]. Because *lag-2::gfp* is expressed in the DTCs of *C. elegans* JK2868, the transgenic nematode *qIs56 [lag-2::gfp]* (JK2868) was used to check the quantity of DTCs after exposure to FAc. After exposure to FAc for 24 h, the fluorescence intensity of DTCs of JK2868 nematodes had no significant difference between the FAc treatment and control (0 mg/L FAc) groups (Figure 3C,D). These results demonstrated that the gonadal development of *C. elegans* was not suppressed by FAc.

### 3.4. FAc Exposure Induced the Germ-Cell Apoptosis of C. elegans

**Figure 4:**
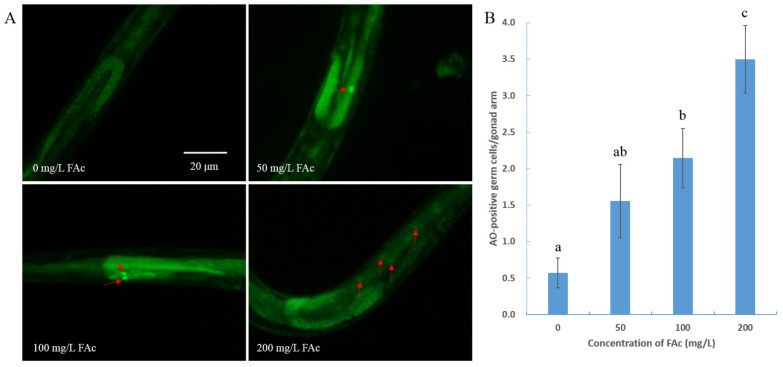
FAc exposure induced the germ-cell apoptosis of *C. elegans*. (**A**) Images of germ cells of *C. elegans*, wild-type young adult worms were treated with FAc in different concentrations for 24 h and stained by using acridine orange (AO). Red arrows indicated apoptotic germ cells. The bar denotes 20 μm. (**B**) Number of apoptotic germ cells of *C. elegans* after exposure to FAc for 24 h. Data are shown as the mean ± standard error (*n* ≥ 7), and different lowercase letters indicate significant difference among treatments by LSD test (*p* < 0.05).

Nematode treatment with active compounds or under stress could trigger germ-cell apoptosis, resulting in a reduction in germ-cell and brood size [23,26,34,38]. To determine whether exposure of FAc could induce the apoptotic response of germ-cell in *C. elegans*, AO staining assay was performed after nematode exposure to FAc for 24 h. As shown in Figure 4, 200 mg/L FAc exposure caused a significant increase in the number of apoptotic germ cells (0.6 at 0 mg/L and 3.5 at 200 mg/L; *p* < 0.001). This FAc-induced germ-cell apoptosis resulted in a dose-dependent increase with increased concentration of FAc from 50 to 200 mg/L (Figure 4B). Thus, FAc exposure significantly increased the level of nematode germ-cell apoptosis.

**Figure 5:**
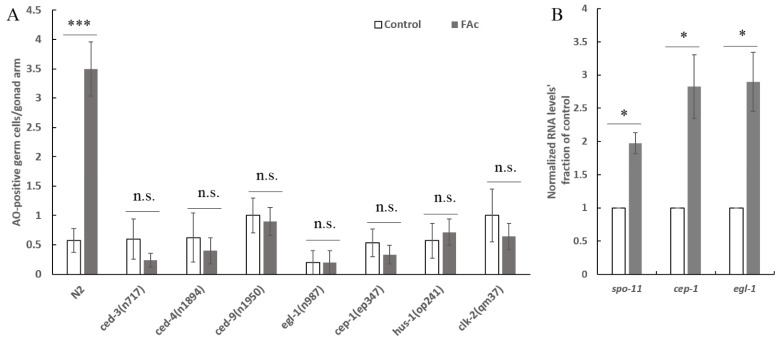
Molecular mechanism of FAc induced the germ-cell apoptosis of *C. elegans*. (**A**) Average number of acridine orange (AO)-positive germ cells per gonad arm in wild-type N_2_ and *C. elegans* mutants after treatment with sterile water (control) or 200 mg/L FAc; data are shown as the mean ± standard error (*n* ≥ 5). (**B**) qPCR analysis of the expression level of genes *spo-11*, *cep-1*, and *egl-1* in wild-type *C. elegans* after treatment with 200 mg/L FAc or sterile water (Control). Gene *pmp-3* served as a control; data are shown as the mean ± standard error (*n* ≥ 3). * *p* < 0.05; *** *p* < 0.001; n.s. not significant; a two-tailed unpaired Student’s *t* test was used for statistical comparison between the values of the treatments and the control.

FAc-induced germ-cell apoptosis was further investigated in *ced-3*, *ced-4*, and *ced-9 C. elegans* mutants, which have mutations in their core apoptotic machinery genes [39,40]. As shown in Figure 5A, the FAc-induced germ-cell apoptosis was abolished in *ced-3*, *ced-4*, and *ced-9 C. elegans* mutants. The p53-like protein CEP-1 reportedly regulates the apoptotic response to DNA damage in germ cells [27,28], and induces its downstream target protein EGL-1 to activate the core apoptosis pathway [26,27,28]. The phenomenon of FAc-induced germ-cell apoptosis was also suppressed by the mutation of gene *cep-1* and its downstream target gene *egl-1* (Figure 5A). CEP-1 has been proposed to be activated by DNA damage checkpoint proteins when protein SPO-11 or other stress induces DNA double-strand breaks [27]. Our results showed that the FAc-induced apoptosis of germ-cell was significantly inhibited when the DNA damage checkpoint protein gene *hus-1* or *clk-2* was mutated (Figure 5A). Moreover, the expression of genes *spo-11*, *cep-1*, and *egl-1* significantly increased after treatment with FAc compared with the control (Figure 5B). Taken together, these results suggested that nematode exposure to FAc might inflict DNA damage through protein SPO-11, and activate the DNA damage checkpoint protein HUS-1 or CLK-2, CEP-1, and EGL-1 in turn, thereby causing germ-cell apoptosis by inducing the core apoptosis pathway.

## 4. Discussion

RKNs are considered as pests of primary importance in agriculture, and the most damaging nematode group in the world [2,41]. As the endoparasites of more than 5500 host plants including rice, wheat, and various vegetables [42], they carry out a part or the entirety of their life cycle in roots to feed and reproduce, causing more than USD 100 billion in annual agriculture losses worldwide [43]. In China, RKN is the most common pathogen in greenhouses; approximately a half of greenhouse-grown vegetables are infected by RKN, and it causes more than USD 400 million in annual losses [44]. RKN control has become a prominent problem in agricultural production. In the present study, FAc, a natural metabolite of bacterial and plant, exhibited RKN control efficiency in pots comparable with that of avermectin (Figure 1, Table 1). FAc was also found to have reproductive inhibition activity on nematodes (Figure 2). Unlike other nematicides with only single nematicidal activity, FAc has various anti-nematode activities, including attract-and-kill effect [18] and inhibition activity of egg-hatching, feeding, and growth [19]. FAc has been proven to inhibit nematodes in all life stages, which can explain why it exhibited a control effect in fields equivalent with those of the commercial nematicides avermectin and metam sodium [19]. FAc has been recognized as a low-toxicity food additive for years [20]. The basic characteristics of FAc combined with the results of this study provide a compelling basis for further investigation of FAc as a potential efficient nematicide.

**Figure 6:**
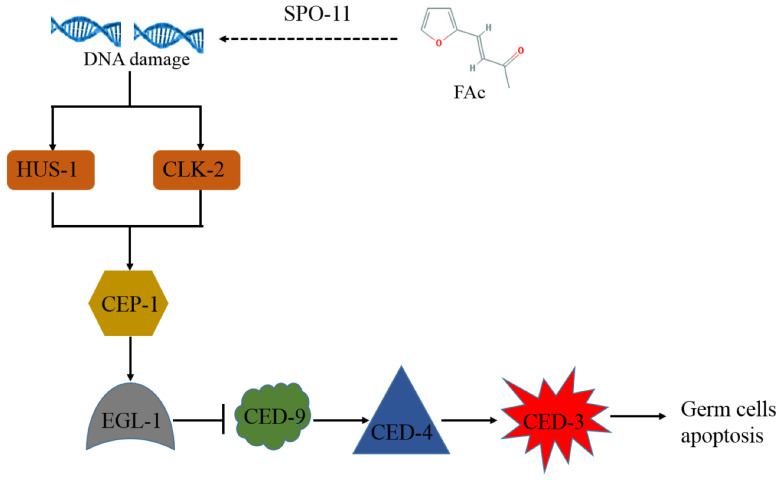
Schematic of model for FAc-induced apoptosis in *C. elegans* germ cells. Nematode exposure to FAc might lead to DNA double-strand breaks through protein SPO-11, and then the DNA damage checkpoint proteins HUS-1 and CLK-2 induce the activation of the p53-like protein CEP-1. CEP-1 regulates the apoptotic response to DNA damage during oogenesis, allowing for the transcription of egl-1. EGL-1 binds with the CED-9 and alleviates CED-9 sequestration of CED-4 to activate CED-3 caspase, leading to germ-cell death.

RKNs reportedly possess strong reproductive ability; a mature female can lay up to 1000 eggs in an egg mass [2]. The large brood size of RKN renders it difficult to control, so identifying drugs with nematode reproductive toxicity is urgent. Many active contents derived from microorganisms or plants have been found to inhibit the activity nematode reproduction [45,46,47]. However, the reproductive-toxicity molecular mechanism of these natural active compounds on nematodes is poorly understood. In the current study, the natural product FAc was found to exhibit reproductive toxicity on *M. incognita* in pots and *C. elegans* in vivo (Figure 2). The model nematode *C. elegans* was used to further investigate the reproductive-toxicity molecular mechanism of FAc on nematodes. The number of germ cells of *C. elegans* was observed to decrease after exposure to FAc (Figure 3A,B), but the DTC fluorescence intensity of nematode treatment with FAc did not significantly change compared with the control (Figure 3C,D). Further investigation revealed that FAc induced the germ-cell apoptosis of *C. elegans* (Figure 4). These results indicated that FAc decreased the brood size of nematodes by inducing germ-cell apoptosis. Results of *C. elegans* mutants AO staining (Figure 5A) and qPCR (Figure 5B) assays revealed that nematode exposure to FAc might cause DNA damage through protein SPO-11, activate CEP-1 and EGL-1, and induce the core apoptosis pathway. Taken together, these results provide a model for FAc-induced apoptosis in *C. elegans* germ cells (Figure 6). After *C. elegans* ingest FAc, it might lead to DNA double-strand breaks through protein SPO-11, and then the DNA damage checkpoint proteins HUS-1 and CLK-2 induce the activation of the p53-like protein CEP-1. CEP-1 regulates the apoptotic response to DNA damage during oogenesis process and transcriptionally induces EGL-1. EGL-1 binds with CED-9 and alleviates the CED-9 sequestration of CED-4 to activate CED-3 caspase, leading to germ-cell death. The germ-cell death of *C. elegans* leads to the decrease in brood size. In this study, a toxic substance, FAc, was found to inflict DNA damage, and induce the core apoptosis pathway to cause germ-cell apoptosis, resulting in decreased brood size of *C. elegans*. However, the current approach in this study also has some limitations, which need to be confirmed by further research. For example, there may be some uncertainties in the location and number of apoptotic cells in *C. elegans* owing to the lack of transgenic nematode *bcIs39 [ced-1::GFP]* (GW1203) for further detection. The discovery of the reproductive-toxicity molecular mechanism of FAc on nematode has primary theoretical value for the subsequent development of efficient nematode control agents. Our findings also broaden the understanding of how microorganisms or plants interact with and combat nematodes through their metabolites.

## Figures and Tables

**Table 1:** Suppression of *M. incognita* by FAc in tomato plants in pot experiment.

Treatment	Dose (mg/pot)	Nematodes in Root/g	*M. incognita* Reduction in Root (%)	Nematodes in Soil/5 g	*M. incognita* Reduction in Soil (%)
FAc ^1^	0	4374 ± 821 a ^3^		75 ± 20 a	
FAc	1	2326 ± 429 b	46.8	38 ± 3 ab	49.6
FAc	2	1775 ± 480 bc	59.4	32 ± 14 b	57.1
Ave ^2^	1	921 ± 306 c	78.9	30 ± 12 b	59.5

^1^ FAc means furfural acetone. ^2^ Ave means avermectin. ^3^ Data are shown as the mean ± standard error (*n* = 5), and different lowercase letters indicate significant difference among treatments by LSD test (*p* < 0.05).
